# Remote Assessment of Post-Stroke Elbow Function Using Internet-Based Telerobotics: A Proof-of-Concept Study

**DOI:** 10.3389/fneur.2020.583101

**Published:** 2020-12-03

**Authors:** Jonghyun Kim, Minki Sin, Won-Seok Kim, Yu-Sun Min, Woojin Kim, Daegeun Park, Nam-Jong Paik, Kyujin Cho, Hyung-Soon Park

**Affiliations:** ^1^School of Mechanical Engineering, Sungkyunkwan University, Suwon, South Korea; ^2^Department of Medical Assistant Robot, Daegu Research Center, Korea Institute of Machinery and Materials, Daegu, South Korea; ^3^Department of Rehabilitation Medicine, Seoul National University College of Medicine, Seoul National University Bundang Hospital, Seongnam, South Korea; ^4^Department of Advanced Robotics, Istituto Italiano di Tecnologia, Genoa, Italy; ^5^School of Mechanical and Aerospace Engineering, Seoul National University, Seoul, South Korea; ^6^Department of Mechanical Engineering, Neurorehabilitation Engineering Lab., Korea Advanced Institute of Science and Technology, Daejeon, South Korea

**Keywords:** telemedicine, telerehabilitation, telerobotics, bilateral haptic feedback, remote assessment, stroke, internet

## Abstract

**Purpose:** Upper limb hemiparesis is the most common impairment in stroke survivors, and adequate assessment is crucial for setting the rehabilitation strategy and monitoring the effect of treatment. However, adequate timely assessments are difficult due to the limited accessibility to clinics for stroke survivors. We designed this study to investigate whether teleassessments for motor impairments of the spastic elbow (i.e., passive range of motion (PROM), muscle strength, and spasticity) are feasible in stroke survivors.

**Methods:** To implement a telerobotic system for remote assessment with physical interaction, we constructed a system with a master robot interacting with a doctor (assessor) and a slave robot interacting with the elbow of a subject with stroke. The master robot is operated by the doctor, where the torque and the speed are transferred to the slave robot via the Internet, and the reaction of the patient's elbow to the slave robot's movement is measured with a torque sensor, then finally transferred back to the master robot. An intercontinental remote assessment, which is considered one of the worst possible scenarios, was used as a clinical test to strictly check the feasibility. For the clinical tests, the examiner for the teleassessment was located at a lab in the National Institutes of Health (NIH, Bethesda, MD, USA) while the stroke patients were located at Seoul National University Bundang Hospital (Bundang, Kyeonggido, South Korea).

**Results:** In total, 12 stroke patients' elbows (age range, 28–74; M:F = 6:6) were tested. For the PROM, the absolute difference between two assessments (in-person vs. remote) was 5.98 ± 3.51° on average (range, 0–11.2). The agreements for the strength and the spasticity of elbow flexor between in-person and remote assessments were substantial (*k* = 0.643) and fair (*k* = 0.308), respectively. No adverse events were observed during or immediately after the telerobotic assessment.

**Conclusions:** Internet-based telerobotic remote assessment for motor impairment of spastic elbow in stroke using our system is feasible even in the worst setting, with too long of a distance and a delayed communication network.

## Introduction

The rapid growth of information and communication technology (ICT) has brought people closer to each other. In healthcare, ICT enables telehealth, which is the remote delivery of health-related services and information (1). Telerehabilitation, a subcomponent of telehealth, is the clinical application of telecommunication technology to provide interventions as well as assessments to patients undergoing rehabilitation in remote locations (2, 3). It can be used to provide cost-effective care and specialized rehabilitation service to patients living far from a rehabilitation center due to the reduced travel time and cost between health centers and the patient's home, as well as direct linkage between the rehabilitation specialist and patient (4). Telerehabilitation will become more important as an increasing number of patients with disabilities require a rehabilitation service in the face of the increasingly aging population as well as the limited accessibility to rehabilitation services due to transportation problems or limited medical staff availability, particularly in rural areas (2). In addition, the current pandemic crisis is revealing the importance of telerehabilitation, which enables the delivery of rehabilitation services without the risk of virus exposure.

Telerehabilitation services can be classified into the two categories of intervention and assessment, both of which are essential. Most studies investigating assessment have attempted to implement video conferencing systems between clinicians in a health center and patients in a remote location such as their home (5). However, these attempts are still far from in-person assessments; for example, all of the assessments lack any physical interaction between clinician and patient (6, 7). Since one of the major targets of rehabilitation is to improve the patient's physical impairments, i.e., plegia, weakness, and spasticity, a clinician needs to assess the impairments by conducting in-person physical exams that involve physical interaction. Without such interaction, the clinician's assessment ability is limited, and the assessment accuracy is degraded (3, 6, 8).

In stroke rehabilitation, comprehensive assessment is essential for proper treatment, quality control, and evaluating training outcomes (9). With the recent advancements in ICT, pilot studies on the telerehabilitation assessment of stroke survivors have been increasing (6). The validity and the reliability of telerehabilitation assessment have mainly been reported in terms of the areas of pain, swelling, the range of motion of joints, muscle strength, balance, gait, posture, special orthopedic testing, and neurodynamic testing, mostly using video conference systems (8, 10–13). The studies mentioned above have two main limitations: The first is that ROM and muscle strength show high validity, whereas posture, special orthopedic testing, and neurodynamic testing have low to moderate validity. Second, video conference systems do not allow for physical interaction between the patient and the evaluator in a remote area (8). Allowing for physical interaction in addition to the video conferencing in telerehabilitation assessment would increase the validity of the assessment. For example, spasticity is a symptom of neurological impairment which is prevalent in patients with stroke, and it plays a very important role in the restoration of function (14). In the recovery of upper limb function after stroke, it is important to accurately evaluate spasticity, particularly elbow spasticity. This is important for evaluating the effectiveness of physical therapy, deciding whether to treat spasticity with Botox injection and medication, and evaluating the effectiveness after treatment (14, 15). However, few studies have evaluated spasticity in telerehabilitation assessment, and the validity of neurodynamic tests is reported to be relatively low (10, 11, 13, 16).

To implement physical interaction between remote locations, a remarkable pilot study for assessing spastic elbow was reported (17). Telerobotics (or bilateral teleoperation) is a concept in the field of robotics which aims to extend the operator's ability (manipulation as well as sensation) to remote areas (18). Since the aim was exactly matched to the required physical interaction for remote assessment, that study attempted to adopt telerobotics technology to provide this interaction. Although the result showed potential usage, it still had several limitations: (1) providing distorted physical interaction due to intuitive robotic devices and control architecture and (2) failure to evaluate the remote assessment due to a limited clinical test with an ideal setup, a small population, and no comparison based on clinical instruments (6). Another study developed a telerobotic device for the remote assessment of hands, but the device was not evaluated in a clinical setup (19).

Hence, the aim of this paper is twofold: improving physical interaction for remote assessment and evaluating that remote assessment. For the former, we developed a novel haptic device to minimize friction that would result in a clinician's inaccurate feeling of the subject's muscle tone, and we applied a control architecture to guarantee stable implementation of physical interaction with time delay that exists in telecommunication, such as the Internet. For the latter, we conducted the clinical test with a challenging setup: an intercontinental Internet-based remote assessment between USA and South Korea. Since time delay is a critical issue in real-time remote assessment, we test this setup as it is the most difficult situation for real-time bilateral physical interaction. Twelve stroke patients with spasticity participated in the clinical test, and typical clinical instruments for the assessment, such as the medical research council scale (MRC) and the modified Ashworth scale (MAS), were used for the comparison between the in-person assessment and the proposed remote assessment.

## Materials and Methods

### Subjects

Twelve stroke patients (six men and six women, 52.6 ± 16.6 years old) with impaired elbows participated in the study (Table 1). They gave written informed consent approved by the Institutional Review Board at SNU Bundang Hospital (IRB approval No.: E1101/058-001).

**Table 1:** Summary of participants (*N* = 12).

	**Sex**	**Age (year)**	**Time since stroke (month)**	**Affected side**
PT01	M	40	1	R
PT02	M	57	3	R
PT03	F	74	7	R
PT04	F	35	29	L
PT05	M	59	54	L
PT06	M	46	30	R
PT07	F	28	48	L
PT08	M	28	37	L
PT09	F	73	27	R
PT10	M	65	92	R
PT11	F	62	49	L
PT12	F	64	27	L

### Instrumentation

#### Telerobotic System for Remote Assessment

**Figure 1:**
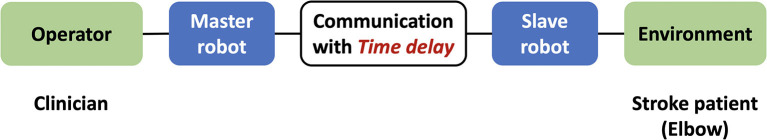
Telerobotic remote assessment.

In robotics, telerobotic systems have often been used to enable physical interaction between the operator and the environment, with the target object in a remote area. As illustrated in Figure 1, such a system consists of a master robot (haptic device) that interacts with the operator, a slave robot that interacts with the environment, telecommunication between the master and the slave, and a control architecture to implement the physical interaction. For the remote elbow assessment, the operator is the clinician who assesses the patient's affected elbow, while the elbow constitutes the environment (Figure 1).

Patients with stroke typically develop several impairments at their affected elbow, such as reduced range of motion (ROM), muscle weakness, and spasticity. Hence, we use the following three tests as the target tasks of the remote assessment: 1) passive ROM test, 2) muscle strength test, and 3) spasticity test. Note that those tests were also used in a previous study on the remote assessment (17).

#### Telerobotic Devices

As mentioned previously, the telerobotic system includes two robotic devices, the master and the slave. During the remote assessment, the clinician must manipulate the master device to move the patient's affected elbow and feel the muscle tone caused by the movement in the elbow. Thus, for accurate assessment, it is crucial for the master device to recreate the resistance (muscle tone) of the affected elbow that the clinician would have felt during an in-person assessment.

**Figure 2:**
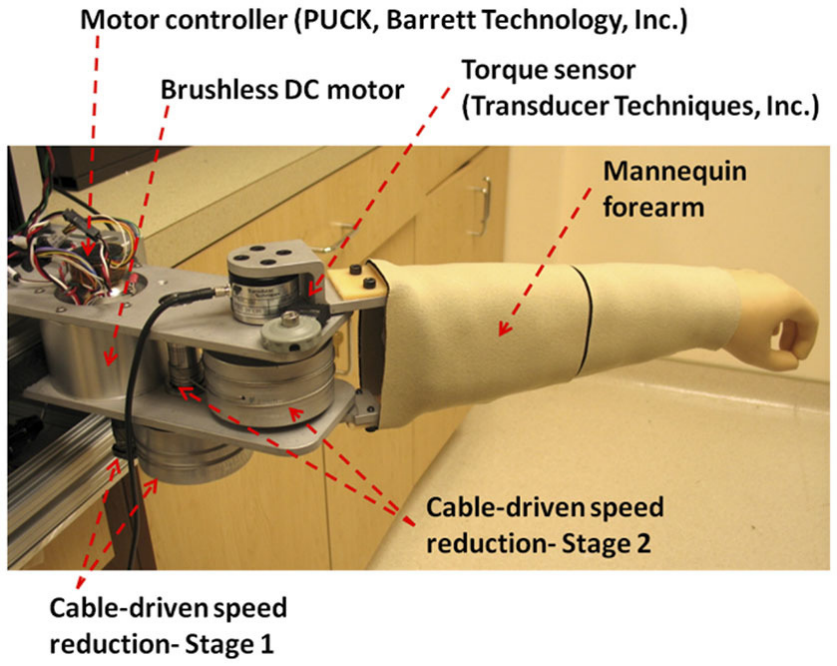
The master robot (clinician site in US).

**Figure 3:**
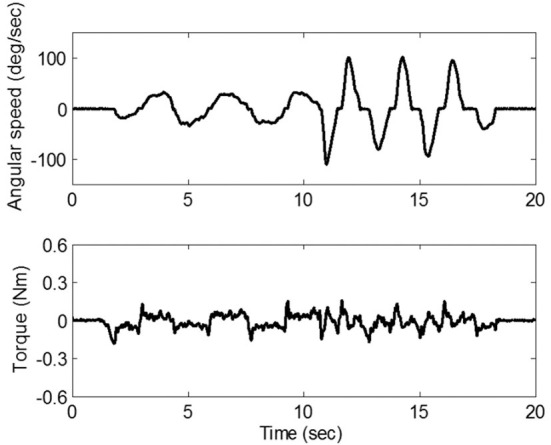
The level of friction due to the cable-driven mechanism in the master device.

In an attempt to reduce the friction, we developed a master device that adopts a cable-driven mechanism, as shown in Figure 2. Through this mechanism, the force/torque generated by a brushless DC motor (Barrett Technology Inc., Cambridge MA) is transmitted to the mannequin arm which mimics the patients' forearm. Since the mechanism utilizes frictionless rolling contact of two adjacent pulleys driven by two steel cables which are pre-tensioned but do not stretch, it can implement a negligible level of friction. To verify the amount of the friction that would be felt by the clinician, we conducted an experiment in which the friction caused by the mechanism was measured using a torque sensor (TRT-200, Transducer Technique Inc., Temecula CA) while the clinician manipulated the mannequin arm with zero motor command. Figure 3 shows that the maximum friction torque was <0.2 Nm, which is small enough to not distort the feel of the resistance due to the affected elbow.

**Figure 4:**
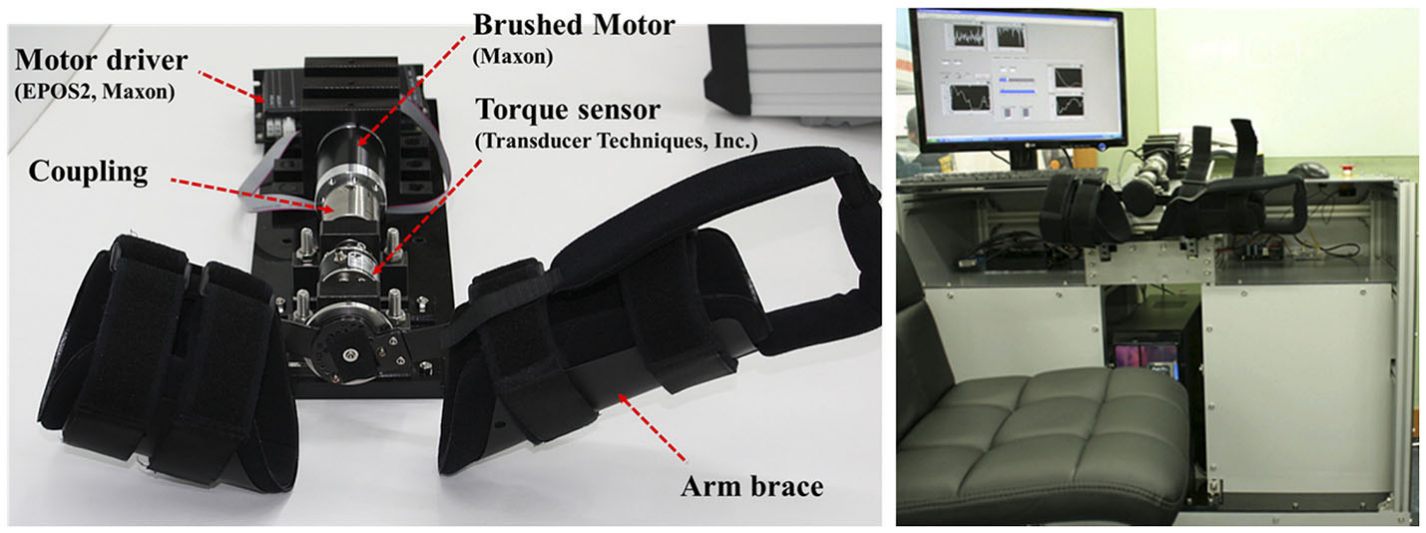
The slave robot (patient site at SNUBH, South Korea).

In addition, for the remote assessment, a slave device is needed to move the elbow by following the command generated by the master and to measure the resistance caused by the affected elbow. We developed an exoskeleton-type slave robot, shown in Figure 4. It contains a brushed DC motor (RE-50, Maxon Motor, Switzerland) to mimic the clinician's movement and a torque sensor (TRT-200, Transducer Technique Inc., Temecula CA) to sense resistance during that movement. The braces in the slave robot were designed to easily attach to and detach from the patient's elbow and to make the patient comfortable while the robot and the patient's forearms move together. The slave robot was equipped with an emergency stop, and the clinician was ready to press the button whenever necessary to ensure safety.

#### Control Architecture

Clinicians need physical interaction with the patients for remote assessment. This can be implemented using a bilateral control architecture that virtually achieves transparent interaction with a certain environment through a telerobotic setup (20). Over the last decade, many control architectures have been reported to provide more transparent interaction, but they have not been able to overcome the well-known conflict between transparency and stability (21, 22); as illustrated in Figure 1, time delay due to communication is unavoidable in a telerobotic system, and it is not easy to achieve stable and transparent interaction under time delay. However, stability is also essential for remote assessment because instability can lead to patients' injuries.

**Figure 5:**
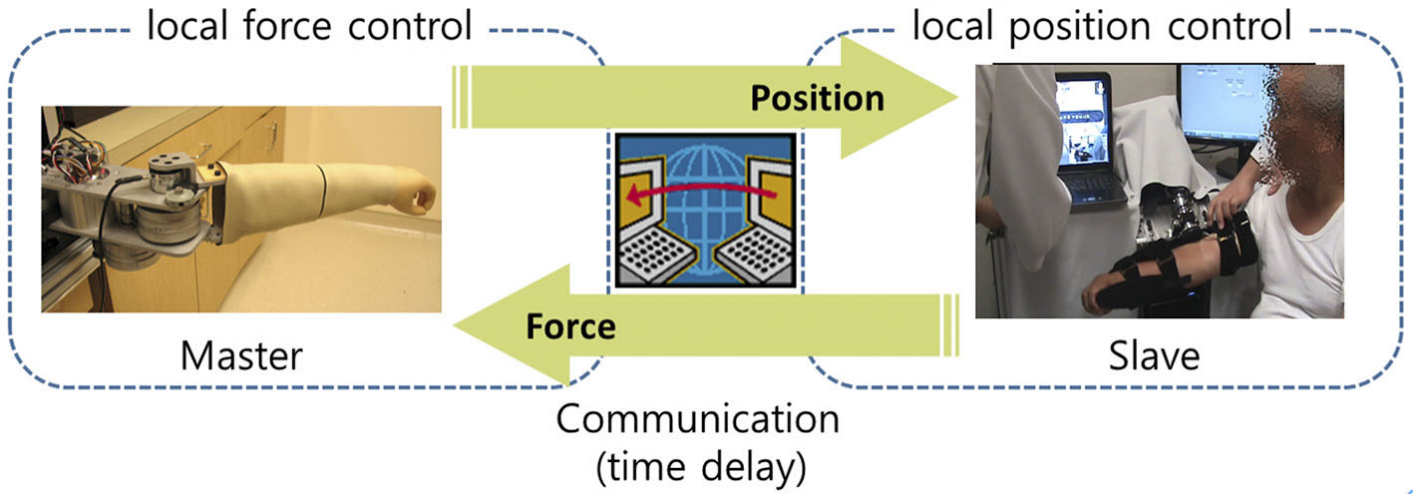
Two-channel F-P control architecture.

A novel control architecture, a two-channel control architecture, has been proposed to overcome this conflict. Since the control architecture provides stable and optimized transparent interaction under a feasible time delay (about up to 500 ms) (23), it can resolve this conflict. Hence, in this paper, we use a two-channel F-P control architecture to implement the physical interaction using master and slave robots. As illustrated in Figure 5, in this architecture, the force of the master followed the transmitted force measured from the slave, and the slave was commanded to follow the position of the master (23). For simplicity, the force controller of the master was not a closed-loop feedback controller but an open-loop feedforward controller. Since the dynamics of the master device can be accurately estimated due to its low friction, the performance of force tracking with the feedforward controller was sufficient to provide appropriate resistance force/torque for the clinician.

To enable communication between the master and the slave, we used the public intercontinental Internet line. The transfer of the current position/force information of the master/slave was implemented with custom-built software using the UDP protocol, which contains checksum to avoid packet corruption/loss for safety. In addition, the Google Hangouts™ application (Google, CA, USA) was used for the video conference between the clinician and the patient.

### Protocol

As mentioned previously, time delay is a critical burden in remote assessment. Therefore, the clinical test for evaluating the assessment was conducted with the worst case we could test: intercontinental Internet-based remote assessment between South Korea and the USA. In this assessment, the master and the slave robots were, respectively, located at the Robotics Laboratory in the National Institutes of Health (Bethesda, MD, USA) and at the SNU Bundang Hospital (Bundang, South Korea). Due to the heavy load and the long distance of the intercontinental Internet line, there was a remarkable time delay of up to 500 ms.

First, a clinician who has 5 years of experience in the hospital examined the subject through passive ROM, muscle strength, and spasticity testing. The clinician used a goniometer to measure the ROM of the subject's affected elbow. The muscle strength was rated according to the medical research council (MRC) score. One inspector measures the ROM and the MRC twice each and used the average of the values. The spasticity of each flexor/extensor was assessed using the modified Ashworth scale (MAS). When measuring the MAS, we conducted the assessment tasks three to five times using the standard protocol (24). The clinician grasped patient's forearm and upper arm and stretched the elbow with a speed of 1 s for full elbow extension. The multiple trials started in a random manner so that the patients could not expect the stretch. The clinician gave MAS score based on the observation of the multiple trials.

**Figure 6:**
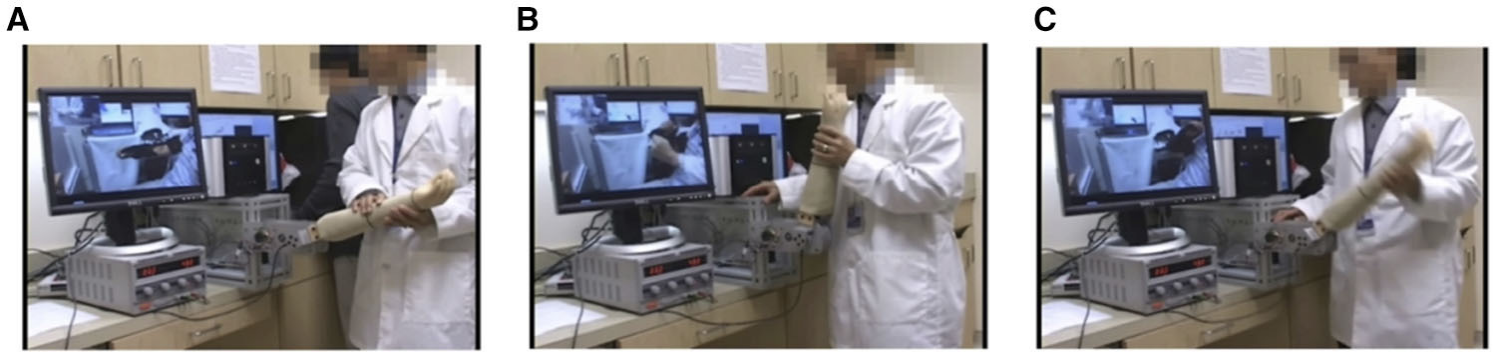
Three remote assessment tasks. **(A)** Passive ROM. **(B)** Muscle strength. **(C)** Spasticity.

After the in-person assessment, the subject was asked to put the slave robot on their affected elbow. The shoulder height of the subject was determined to a natural posture where the shoulder girdle is not elevated or depressed. The elbow joint axis was aligned with the slave robot axis by moving the forearm while wearing the slave robot and adjust the elbow joint position that does not make any slide motion with the arm and brace part of the slave robot. After that, the subject's neutral joint position was determined manually by the clinician using a goniometer and the angle of the master device was synchronized with the slave device at the neutral joint position to eliminate the angle difference. Next, the following three remote assessment tasks were carried out by a clinical staff who has 6 years of experience in spasticity, muscle strength, and range of motion assessment, as shown in Figure 6.

1) Passive ROM test: At the beginning of the assessment session, the clinician slowly moved the mannequin arm at the master device. This commanded the slave device, through the Internet, to move the subject's elbow in the same way, and the resistance torque at the subject's elbow was recreated in the master device to provide real-time haptic feeling of the subject's elbow joint to the clinician. Due to this haptic feedback, the clinician could remotely detect the position limits in both elbow flexion and extension under controlled peak resistance torque. This test was taken twice, and the ROM was determined based on the minimum and maximum angles measured during the test (Figure 6A).
2) Muscle strength test: Using the video conferencing tool, the clinician asked the subject to flex/extend his or her elbow while the clinician remotely held the elbow at a selected position. The slave device simply held the subject's elbow according to the position of the master device, and the subject repeated three flexion and extension motion in isometric conditions with 15 s rest between each trial. The measured torque generated by the subject was sent to the master device and the clinician felt the torque generated by the subject during the test, then rated the MRC score (Figure 6B).
3) Spasticity test: Spasticity was evaluated remotely by moving the subject's elbow through the ROM determined above. The resulting “muscle tone” was felt by the clinician remotely, which allowed the clinician to make a determination about the MAS score, a measure of the spasticity. The passive movement was done three to five times in both flexion and extension as well as at several velocities to determine MAS score clearly, simulating those in clinical examinations (Figure 6C).

### Data Analysis

To evaluate the proposed remote assessment, the clinical outcomes of the assessment were compared with those of in-person assessment. The ROM error, defined as the difference of ROM between in-person and remote conditions, was calculated to evaluate the passive ROM test. For muscle strength and spasticity, the agreement between in-person and remote assessments was evaluated using Cohen's kappa statistics.

## Results

### Passive ROM Test

**Figure 7:**
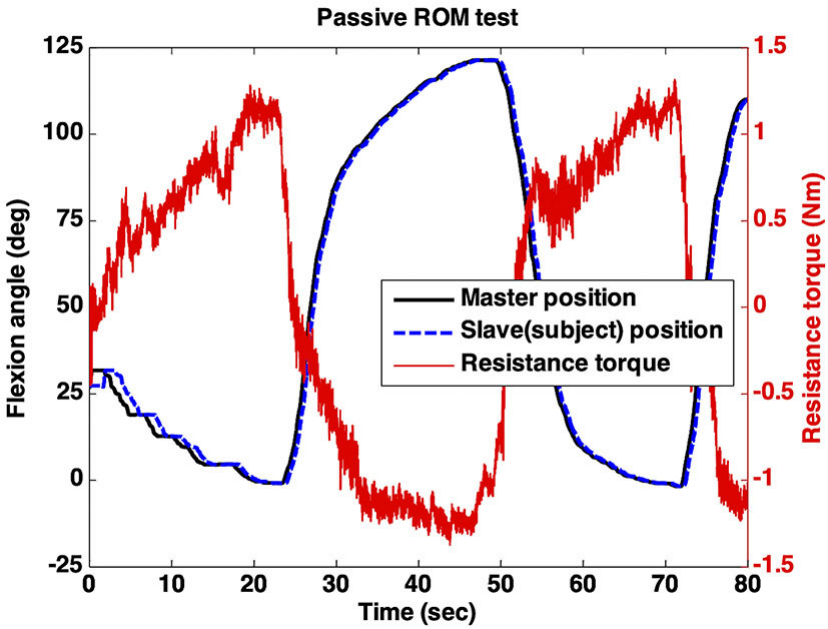
Remote passive ROM test.

Figure 7 shows a representative passive ROM test that was remotely conducted using the telerobotic device. As shown in the figure, the slave position (blue line) followed the master position (black line) with a time delay. For instance (PT11), as shown in Figure 7, the clinician started to move the master at the initial position and stopped at a near-zero flexion angle (0.5°) due to increased resistance torque. As a result, the position limit of elbow extension was determined to be 0.5°. Next, the master was flexed by the clinician again while significant resistance torque appeared. Therefore, the position limit of elbow flexion was determined to be the end position (122.5°) measured by the encoder attached to the master. Those two position limits result in an outcome of remote assessment indicating 122° passive ROM of the subject's affected elbow.

The passive ROMs obtained by remote assessment were compared with those obtained by in-person assessment, and the comparison is presented in Table 2. The absolute differences between the two ROMs were <6° on average (5.98 ± 3.51°).

**Table 2:** Comparison of outcomes (PROM, MRC, and MAS) between in-person and remote assessments.

	**Passive ROM (deg)**		**MRC scale**		**MAS**	
	**In-person**	**Remote**	**In-person**	**Remote**	**In-person**	**Remote**
PT01	130	140.4	5	5	0	0
PT02	130	131.8	3	3	1+	1+
PT03	134	125.8	4	4	0	0
PT04	130	131.5	5	5	2	0
PT05	135	130.1	4	4	2	1
PT06	120	131.2	5	5	1+	0
PT07	140	140.0	4	3	1	0
PT08	141	135.7	4	4	2	2
PT09	111	118.7	3	2	2	2
PT10	130	121.5	5	5	1	2
PT11	128	122.0	3	3	2	2
PT12	134	127.7	4	3	1+	2

### Muscle Strength Test

**Figure 8:**
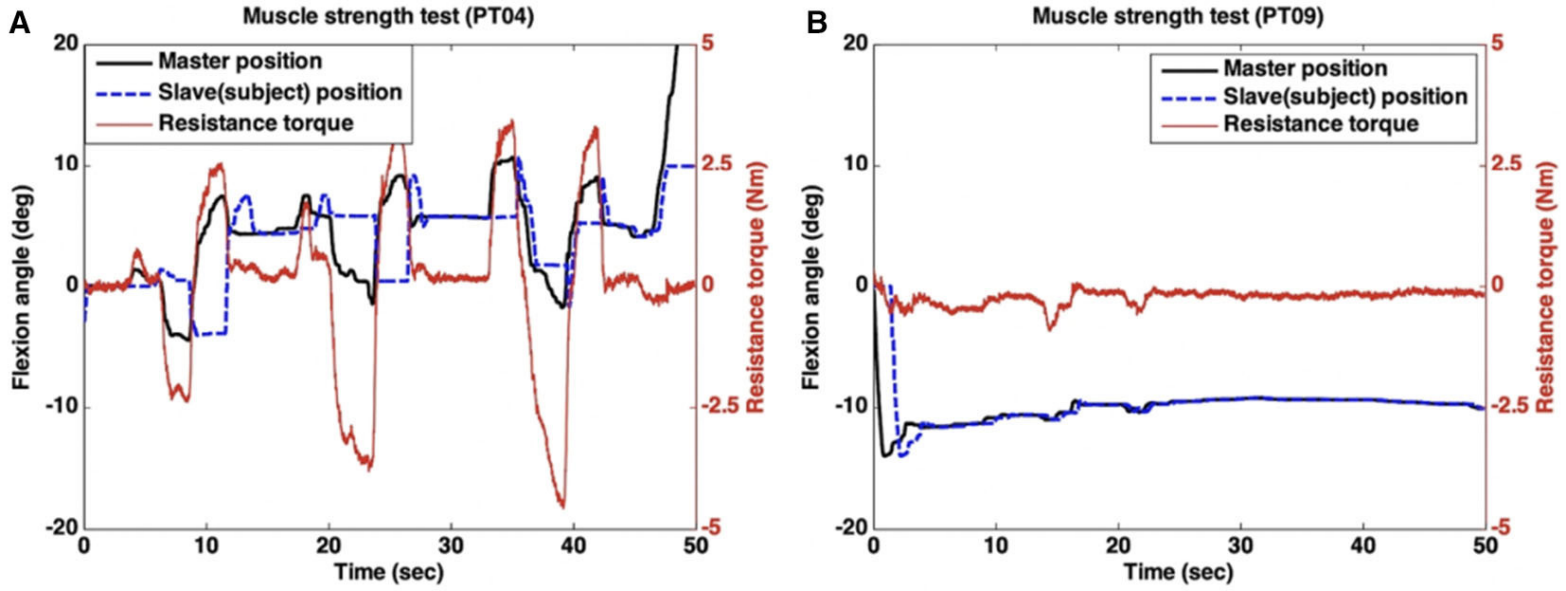
Remote muscle strength test. **(A)** MRC3 case, **(B)** MRC5 case.

In this test, the subjects did their best to maintain the position (flexion angle) shown in Figure 6B against the force applied remotely by the clinician. Figure 8 shows the data of two representative subjects in the proposed remote assessment setup. One subject with good muscle strength (MRC 5) was able to generate large resistance torque to compensate for the force that was applied by the clinician, as displayed in Figure 8A. By contrast, the other subject (MRC 3) achieved negligible resistance torque against the clinician's movement (Figure 8B).

The MRC scales rated by in-person assessment and remote assessment are summarized in Table 2. The results show substantial agreement between the two MRC scales (*k* = 0.643).

### Spasticity Test

**Figure 9:**
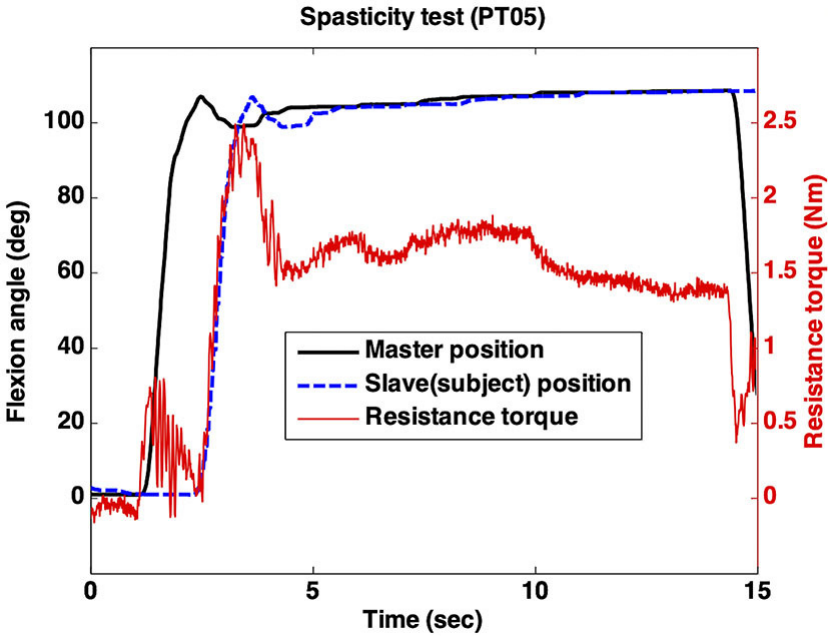
Remote spasticity test.

In this test, the clinician manipulated the master device quickly, as displayed in Figure 6C. This fast movement resulted in a rapid increase in the resistance torque caused by the subject's impaired elbow. MAS 1 indicates a small but rapid increase in the resistance torque. Since a large and rapid increase appeared and remained at almost the total ROM of the elbow, MAS was determined to be 2 (Figure 9).

As summarized in Table 2, the MAS rated by in-person and remote assessments only had fair agreement (*k* = 0.308). The clinician rated the MAS according to the size of the increase and the start/end positions of the increased torque. As shown in Figure 9, the clinician most felt the increase at the end of ROM of the elbow due to the time delay. Hence, the start/end positions of the increased torque were not clear.

## Discussion

This paper implemented and tested remote real-time physical assessment. Considering that physical assessment involves haptic feel between the examiner and examinee, it would be more realistic to implement haptic interaction at remote locations through the use of telerobotic control technology. Using a teleoperated robot in the rehabilitation field may help cost-effectively solve the accessibility issues involved when a patient and a rehabilitation specialist are not able to meet face to face. For example, rehabilitation service access may be limited in patients living in areas far from rehabilitation facilities (25). In this situation, simple instructions and training given to the person who will manage the slave robot may be sufficient to provide telerehabilitation operated by a remotely located rehabilitation specialist, without travel. This clinical scenario is also relevant to developing countries that have limited rehabilitation human resources (26) and isolated conditions in some regions and medical or nursing facilities due to outbreaks of communicable diseases. In situations like the recent COVID-19 outbreak, this system may be helpful for protecting the therapist in intrahospital rehabilitation services.

Although the 1 DoF robotic system in this study is limited to use in arm rehabilitation after central nervous system injury, this concept of a teleoperated robot with haptic feeling can also be applied to a multidimensional rehabilitation robot, which may be useful for telerehabilitation beyond teleassessments (27). Our system is also promising as it may provide education to potential rehabilitation therapists in developing countries. Knowledge transfer is possible through lectures using video conferencing, but hands-on education is necessary for rehabilitation training. Therefore, education with a teleoperating haptic robotic system provided by an experienced therapist in developed countries may be more effective than online lectures alone. This tele-education may also be more cost-effective than onsite education with a visit by a trained therapist, considering cost for travel, accommodation, and travel time (indirect cost).

Due to the control architecture and low friction of the master robot, the performance of a real-time teleassessment system mainly relies on the time delay between the examiner and examinee. According to the teleoperation control theory (21), the time delay degrades the quality of the haptic feel and the stability of the robotic systems. We have tested our experimental setup under a maximum time delay of 500 ms, and the performances of the passive ROM and the muscle strength test were accurate; specifically, the joint movements and the measured torques of the master and the slave robots were similar. This was possible because the movement speed of those tasks was slow enough to not be affected by the maximum time delay; however, the spasticity assessment was conducted with a fast speed (maximum stretching speed of 210°/s), and the time delay significantly degraded the haptic performance. It is possible that the amount of time delay can be significantly reduced, and we hope that this would improve the performance of fast tasks. With 5G technology being implemented worldwide, data transfer speeds are expected to increase. In addition, considering that we had to use a virtual private network to deal with the Internet security issue at the hospital, the data transfer could be faster if the hospital were to open an Internet gateway allowing for a direct connection between the two places.

The disagreement in the remote spasticity assessment could also come from the characteristics of MAS, the measure of spasticity used. In contrast to ROM and MRC, the inter-rater reliability of MAS has been known to range from poor to fair (28, 29). The MAS scores from the in-person assessment and the teleassessment had fair agreement in this study (*k* = 0.308), and this was within the range of the reported inter-rater reliability of the MAS scoring system (30, 31). Hence, two clinicians who participated in South Korea and the USA could rate different MAS even though the telerobotic system provides ideal haptic performance.

In this study, we did not evaluate the test-retest and inter-rater reliabilities of the proposed remote assessment. We expect that the reliabilities would be comparable with in-person assessment, but the effect of degraded haptic feeling with different raters must be investigated. There was no qualitative evaluation (i.e., questionnaires on satisfaction) of the proposed assessment compared with in-person assessment. Finally, the proposed assessment was verified with small numbers of clinicians and stroke patients. Therefore, further research including cost analysis with larger sample sizes is required.

## Data Availability Statement

The raw data supporting the conclusions of this article will be made available from the corresponding authors on request.

## Ethics Statement

The studies involving human participants were reviewed and approved by Institutional Review Board at Seoul National University Bundang Hospital (IRB approval No.: E1101/058-001). The patients/participants provided their written informed consent to participate in this study.

## Author Contributions

H-SP, KC, and N-JP conceived the study idea and coordinated the study. JK, MS, and DP developed the telerobotic system. H-SP and KC supervised the engineering development. H-SP, JK, MS, Y-SM, DP, and WK involved in the experiments. JK, Y-SM, and WK analyzed the data. JK and W-SK drafted the manuscript, and all authors revised the manuscript.

## Conflict of Interest

The authors declare that the research was conducted in the absence of any commercial or financial relationships that could be construed as a potential conflict of interest.

## References

[1] Zonneveld M Patomella A-H Asaba E Guidetti S. The use of information and communication technology in healthcare to improve participation in everyday life: a scoping review. Disabil Rehabil. (2019) 2019:1–8. 10.1080/09638288.2019.1592246 30966833

[2] Brennan DM Mawson S Brownsell S. Telerehabilitation: enabling the remote delivery of healthcare, rehabilitation, self management. Stud Health Technol Inform. (2009) 145:231–48. 10.3233/978-1-60750-018-6-231 19592797

[3] Rogante M Grigioni M Cordella D Giacomozzi C. Ten years of telerehabilitation: a literature overview of technologies and clinical applications. NeuroRehabilitation. (2010) 27:287–304. 10.3233/NRE-2010-0612 21160118

[4] Bernard M-M Janson F Flora P Faulkner G Meunier-Norman L Fruhwirth M Videoconference-Based physiotherapy and tele-Assessment for homebound older adults: a pilot study. Adapt Aging. (2009) 2009:39–48. 10.1080/01924780902718608

[5] Laver KE Schoene D Crotty M George S Lannin NA Sherrington C. Telerehabilitation services for stroke. Cochr Datab Syst Rev. (2013) 2013:CD010255. 10.1002/14651858.CD010255.pub2 24338496 PMC6464866

[6] Appleby E Gill ST Hayes LK Walker TL Walsh M Kumar S. Effectiveness of telerehabilitation in the management of adults with stroke: a systematic review. PLoS ONE. (2019) 14:e225150. 10.1371/journal.pone.0225150 31714924 PMC6850545

[7] Navarro E González P López-Jaquero V Montero F Molina JP Romero-Ayuso D. Adaptive multisensorial, physiological and social: the next generation of telerehabilitation systems. Front Neuroinformatics. (2018) 12:43. 10.3389/fninf.2018.00043 30042671 PMC6049338

[8] Mani S Sharma S Omar B Paungmali A Joseph L. Validity and reliability of Internet-based physiotherapy assessment for musculoskeletal disorders: a systematic review. J Telemed Telecare. (2017) 23:379–91. 10.1177/1357633X16642369 27036879

[9] Duncan PW Horner RD Reker DM Samsa GP Hoenig H Hamilton B. Adherence to postacute rehabilitation guidelines is associated with functional recovery in stroke. Stroke. (2002) 33:167–77. 10.1161/hs0102.101014 11779907

[10] Lade H McKenzie S Steele L Russell TG. Validity and reliability of the assessment and diagnosis of musculoskeletal elbow disorders using telerehabilitation. J Telemed Telecare. (2012) 18:413–8. 10.1258/jtt.2012.120501 23086982

[11] Truter P Russell T Fary R. The validity of physical therapy assessment of low back pain via telerehabilitation in a clinical setting. Telemed E-Health. (2013) 20:161–7. 10.1089/tmj.2013.0088 24283249

[12] Palacín-Marín F Esteban-Moreno B Olea N Herrera-Viedma E Arroyo-Morales M. Agreement between telerehabilitation and face-to-face clinical outcome assessments for low back pain in primary care. Spine. (2013) 38:947–52. 10.1097/BRS.0b013e318281a36c 23238489

[13] Russell TG Blumke R Richardson B Truter P. Telerehabilitation mediated physiotherapy assessment of ankle disorders. Physiother Res Int. (2010) 15:167–75. 10.1002/pri.471 20812313

[14] Francisco Gerard E McGuire John R. Poststroke spasticity management. Stroke. (2012) 43:3132–6. 10.1161/STROKEAHA.111.639831 22984012

[15] Dong Y Wu T Hu X Wang T Efficacy and safety of botulinum toxin type A for upper limb spasticity after stroke or traumatic brain injury: a systematic review with meta-analysis and trial sequential analysis. Eur J Phys Rehabil Med. (2017) 53:256–67. 10.23736/S1973-9087.16.04329-X 27834471

[16] Steele L Lade H McKenzie S Russell TG. Assessment and diagnosis of musculoskeletal shoulder disorders over the internet. Int J Telemed Appl. (2012) 2012:e945745. 10.1155/2012/945745 23193395 PMC3501948

[17] Park H-S Peng Q Zhang L-Q. A portable telerehabilitation system for remote evaluations of impaired elbows in neurological disorders. IEEE Trans Neural Syst Rehabil Eng Publ IEEE Eng Med Biol Soc. (2008) 16:245–54. 10.1109/TNSRE.2008.920067 18586603

[18] Avgousti S Christoforou EG Panayides AS Voskarides S Novales C Nouaille L. Medical telerobotic systems: current status and future trends. Biomed Eng OnLine. (2016) 15:96. 10.1186/s12938-016-0217-7 27520552 PMC4983067

[19] Kim J-Y Park G Lee S-A Nam Y. Analysis of machine learning-Based assessment for elbow spasticity using inertial sensors. Sensors. (2020) 20:1622 10.3390/s20061622 32183281 PMC7146614

[20] Lawrence DA Stability and transparency in bilateral teleoperation. IEEE Trans. Robot. Autom. (1993) 9:624–37. 10.1109/70.258054

[21] Hashtrudi-Zaad K Salcudean SE Transparency in time-delayed systems and the effect of local force feedback for transparent teleoperation. IEEE Trans Robot Autom. (2002) 18:108–14. 10.1109/70.988981

[22] Chang PH Kim J. Telepresence index for bilateral teleoperations. IEEE Trans Syst. (2012) 42:849. 10.1109/TSMCB.2011.2160849 21824852

[23] Kim J Chang PH Park H-S. Two-channel transparency-optimized control architectures in bilateral teleoperation with time delay. IEEE Trans Control Syst Technol. (2013) 21:40–51. 10.1109/TCST.2011.2172945 23833548 PMC3702193

[24] Bohannon RW Smith MB. Interrater reliability of a modified ashworth scale of muscle spasticity. Phys Ther. (1987) 67:206–7. 10.1093/ptj/67.2.206 3809245

[25] Centers for Disease Control and Prevention. Outpatient rehabilitation among stroke survivors-21 States and the District of Columbia 2005. MMWR Morb Mortal Wkly Rep. (2007) 56:504–7. 17522589

[26] Brainin M Teuschl Y Kalra L. Acute treatment and long-term management of stroke in developing countries. Lancet Neurol. (2007) 6:553–61 10.1016/S1474-4422(07)70005-4 17509490

[27] Baur K Rohrbach N Hermsdörfer J Riener R Klamroth-Marganska V. The “Beam-Me-In Strategy” -remote haptic therapist-patient interaction with two exoskeletons for stroke therapy. J Neuroengin Rehab. 16:85. 10.1186/s12984-019-0547-3 31296226 PMC6625018

[28] Park H-S Kim J Damiano DL. Development of a Haptic Elbow Spasticity Simulator (HESS) for improving accuracy and reliability of clinical assessment of spasticity. IEEE Trans. Neural Syst. Rehab. Engin. (2012) 20:5330. 10.1109/TNSRE.2012.2195330 22562769 PMC3579668

[29] Choi S Shin YB Kim S-Y Kim J. A novel sensor-based assessment of lower limb spasticity in children with cerebral palsy. J NeuroEngin Rehab. (2018) 15:45. 10.1186/s12984-018-0388-5 29866177 PMC5987429

[30] Sunnerhagen KS. Stop using the Ashworth scale for the assessment of spasticity. J Neurol Neurosurg Psychiatry. (2010) 81:2. 10.1136/jnnp.2009.189068 20019216

[31] Sung J Choi S Kim J Kim J. Simplified estimation of abnormal reflex torque due to elbow spasticity using neuro-musculoskeletal model. Annu Int Conf IEEE Eng Med Biol Soc. (2019) 2019:5076–9. 10.1109/EMBC.2019.8856613 31947000

