# Corrigendum: Remote Assessment of Post-Stroke Elbow Function Using Internet-Based Telerobotics: A Proof-of-Concept Study

**DOI:** 10.3389/fneur.2020.640537

**Published:** 2021-02-04

**Authors:** Jonghyun Kim, Minki Sin, Won-Seok Kim, Yu-Sun Min, Woojin Kim, Daegeun Park, Nam-Jong Paik, Kyujin Cho, Hyung-Soon Park

**Affiliations:** ^1^School of Mechanical Engineering, Sungkyunkwan University, Suwon, South Korea; ^2^Department of Medical Assistant Robot, Daegu Research Center, Korea Institute of Machinery and Materials, Daegu, South Korea; ^3^Department of Rehabilitation Medicine, Seoul National University College of Medicine, Seoul National University Bundang Hospital, Seongnam, South Korea; ^4^Department of Advanced Robotics, Istituto Italiano di Tecnologia, Genoa, Italy; ^5^School of Mechanical and Aerospace Engineering, Seoul National University, Seoul, South Korea; ^6^Department of Mechanical Engineering, Neurorehabilitation Engineering Lab., Korea Advanced Institute of Science and Technology, Daejeon, South Korea

**Keywords:** telemedicine, telerehabilitation, telerobotics, bilateral haptic feedback, remote assessment, stroke, internet

Daegeun Park was not included as an author in the published article. The corrected author list, affiliations, and Author Contributions Statement appear below.

Corrected author list and affiliations:

Jonghyun Kim^1†^, Minki Sin^2†^, Won-Seok Kim^3†^, Yu-Sun Min^3^, Woojin Kim^3^, Daegeun Park^4^, Nam-Jong Paik^3^^*^, Kyujin Cho^5^^*^, Hyung-Soon Park^6^^*^

^1^ School of Mechanical Engineering, Sungkyunkwan University, Suwon, South Korea

^2^ Department of Medical Assistant Robot, Daegu Research Center, Korea Institute of Machinery and Materials, Daegu, South Korea

^3^ Department of Rehabilitation Medicine, Seoul National University College of Medicine, Seoul National University Bundang Hospital, Seongnam, South Korea

^4^ Department of Advanced Robotics, Istituto Italiano di Tecnologia, Genoa, Italy

^5^ School of Mechanical and Aerospace Engineering, Seoul National University, Seoul, South Korea

^6^ Department of Mechanical Engineering, Neurorehabilitation Engineering Lab., Korea Advanced Institute of Science and Technology, Daejeon, South Korea

## Author Contributions

H-SP, KC, and N-JP conceived the study idea and coordinated the study. JK, MS, and DP developed the telerobotic system. H-SP and KC supervised the engineering development. H-SP, JK, MS, Y-SM, DP, and WK involved in the experiments. JK, Y-SM, and WK analyzed the data. JK and W-SK drafted the manuscript, and all authors revised the manuscript.

